# Small Nucleolar RNAs and the Brain: Growing Evidence Supporting Their Role in Psychiatric Disorders

**DOI:** 10.1016/j.bpsgos.2024.100415

**Published:** 2024-11-10

**Authors:** Juliette Salles, Rixing Lin, Gustavo Turecki

**Affiliations:** a McGill Group for Suicide Studies, Douglas Mental Health University Institute, Department of Psychiatry, McGill University, Montreal, Quebec, Canada; b Princeton Neuroscience Institute, Princeton University, Princeton, New Jersey

**Keywords:** Brain, Human, Noncoding RNAs, Pathophysiological mechanisms, Psychiatric disorders, Small nucleolar RNAs

## Abstract

Noncoding RNAs comprise most of the transcriptome and represent an emerging area of research. Among them, small nucleolar RNAs (snoRNAs) have emerged as a promising target because they have been associated with the development and evolution of several diseases, including psychiatric disorders. snoRNAs are expressed in the brain, with some showing brain-specific expression that indicates specific roles in brain development, function, and dysfunction. However, the role of snoRNAs in conditions that affect the brain needs further investigation to be better understood. This scoping review summarizes existing literature on studies that have investigated snoRNAs in psychiatry and offers insight into potential pathophysiological mechanisms to be further investigated in future research.

## Plain Language Summary

Small nucleolar RNAs (snoRNAs) have emerged as a promising target for investigation because they have been associated with the development and evolution of several diseases, including psychiatric disorders. In this scoping review, we summarize existing literature on studies investigating snoRNAs in psychiatry and offer insight into potential pathophysiological mechanisms to be further investigated in future research.

## Small Nucleolar RNA Expression and Roles

Most of the transcriptome of the genome consists of noncoding RNAs (ncRNAs) (1). NcRNAs can be divided into different classes, broadly based on their size (2) or their functions (3,4).

**Figure 1:**
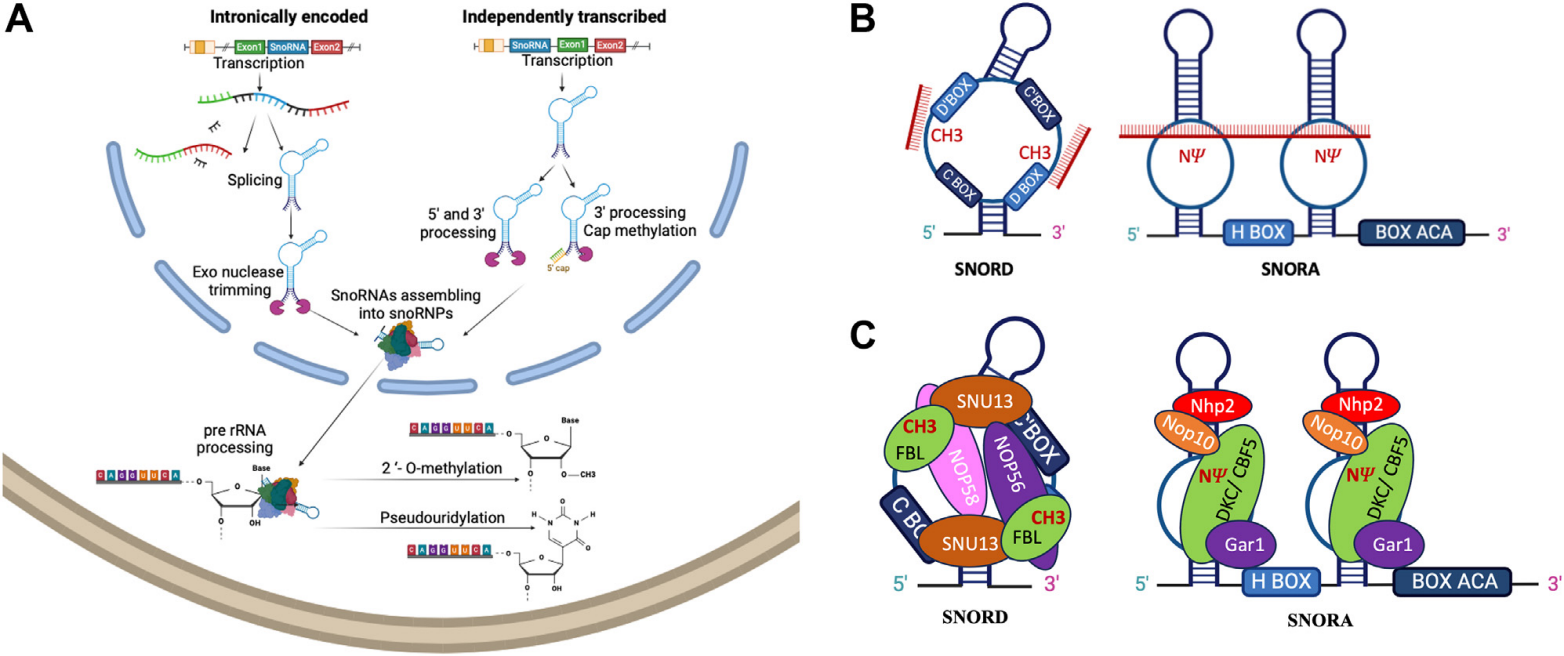
**(A)** Transcription, processing. snoRNA genes are predominantly located within gene introns and released from the host gene precursor messenger RNA by splicing (5); the expression can be regulated independently from the level of the host RNA or other snoRNAs within the same transcription unit (4). RNA polymerase II transcribes the majority of independently transcribed snoRNA genes, but most of the information regarding the promoters of these genes comes from yeast (96), and the structure of promoters is largely unknown in humans although some progress has been made through bioinformatic analysis (97). Once their host intron is excised, the snoRNAs are embedded in RNAs without a 5′ cap or a polyA tail. snoRNA may also be associated to long noncoding RNAs; some of them are 5′ snoRNA capped and 3′ polyadenylated carrying a snoRNA at both ends or only at the 5′ end, respectively (98). These snoRNAs protect the inner sequence from degradation, thus creating long noncoding RNA–snoRNA hybrids (99). During transcription, the 5′ m7G cap is cotranscriptionally added to the nascent molecule and then removed (100) or converted to a TMG cap (101). A 3′ end processing is also required for final maturation. **(B)** Overview of structural canonical SNORD with C box and a D box motif located close to the terminal stem and additional internal C' and D' boxes (location of methylation from Box D/D’) and canonical SNORA with 2 stem loop structures with an internal H box motif and an ACA box motif at the 3′ end (location of pseudouridylation [NΨ]) at the base of the hairpins). **(C)** Assemblage of protein factors on the snoRNA (boxes C/D and H/ACA snoRNPs) that protect snoRNAs from exonucleolytic degradation (102). rRNA, ribosomal RNA; SNORA, H/ACA box snoRNA; SNORD, C/D box snoRNA; snoRNA, small nucleolar RNA; snoRNP, snoRNA-ribonucleoprotein complex.

Small nucleolar RNAs (snoRNAs) belong to the class of intermediate size (60- to 300-nucleotide-long) ncRNAs. They are found in all eukaryotic genomes, even if their organization and regulation has changed considerably throughout evolution (5), and their number increases with the complexity of the organism. For example, in the yeast *Saccharomyces cerevisiae*, 64 transcription units encode 76 snoRNAs, and 731 reads are found in *Potamochoerus porcus*, whereas in humans, a recent update of the snoRNA database lists 2123 predicted snoRNAs (6), 505 of which have been validated by sequencing in human skeletal muscle, liver, testis, and brain tissues (7,8). In addition, snoRNAs could be located in unfavorable loci, defined by the absence of a nearby active promoter and/or the absence of flanking sequences that allow the formation of a terminal stem. This explains why most annotated vertebrate snoRNAs are not expressed; in fact, most vertebrates are predicted to have only between 13% and 45% of expressed snoRNAs (9). Among the expressed snoRNAs, only a small fraction of the snoRNA gene is not inserted in the host gene and is expressed from independent promoters. The process of snoRNA transcription is shown in Figure 1A. Their canonical function is to facilitate the pseudouridylation and 2′-*O*-methylation of ribosomal RNAs (rRNAs) (10,11), as described in the Supplement.

snoRNAs are divided in 2 main classes: the C/D box snoRNAs (SNORDs) and the H/ACA box snoRNAs (SNORAs). SNORDs are characterized by the presence of 2 conserved sequence motifs, box C (UGAUGA) and box D (CUGA), near the 5′ and 3′ termini, respectively (Figure 1B). snoRNAs associate with protein to form snoRNA-ribonucleoprotein complexes (Figure 1C). snoRNA-ribonucleoprotein complex associations and function are described in the Supplement.

Most snoRNAs are expressed uniformly across tissues, while 18% of snoRNAs are enriched in specific tissues (7). Interestingly, the most tissue-specific snoRNAs are found in the brain and reproductive tissues; 10% of the snoRNAs are enriched in the brain (Box 1) (7). Another study noted that the evolutionary changes in frontal cortex expression of snoRNAs across humans, chimpanzees, rhesus monkeys, and mice shows a substantial divergence of snoRNA expression levels among the 4 mammalian species at the individual gene level. Of the 183 snoRNA genes present in the 4 species and expressed in brain, 83 (44%) showed divergent expression among species, but the bias toward evolutionary acceleration in the human evolutionary lineage was moderate (12). However, the example of the divergence of *SNORA29* snoRNA expression in the human lineage is particularly striking. *SNORA29* maintains high expression levels in mice, macaques, and chimpanzees but decreases significantly in humans. This decrease could be caused by the reduced stability of its secondary structure. However, the authors specified that these results do not provide information about the role of *SNORA29* in the evolution of human-specific cognitive features (12). As with other RNA types, the degree of snoRNA expression divergence among species is largely proportional to phylogenetic divergence. This may suggest that snoRNA expression divergence merely reflects the accumulation of genetic divergence between species and has no functional effect. However, this hypothesis is difficult to reconcile with the essential role of snoRNA in modifying highly conserved functional RNA molecules. Therefore, the authors proposed that brain snoRNAs play a more dynamic role in RNA-based regulatory and functional networks than is currently recognized (12).

Box 1 **Brain Enriched snoRNAs**

**Table:** 

SNORD3	SNORD115-15	SNORD115-25	SNORD115-36	SNORD115-45
SNORD108	SNORD115-16	SNORD115-26	SNORD115-37	SNORD115-48
SNORD109B	SNORD115-17	SNORD115-29	SNORD115-38	SNORD115-5
SNORD115	SNORD115-19	SNORD115-3	SNORD115-39	SNORD115-6
SNORD115-1	SNORD115-2	SNORD115-30	SNORD115-4	SNORD115-8
SNORD115-10	SNORD115-20	SNORD115-31	SNORD115-40	SNORD115-9
SNORD115-11	SNORD115-21	SNORD115-32	SNORD115-41	SNORA35
SNORD115-12	SNORD115-22	SNORD115-33	SNORD115-42	
SNORD115-13	SNORD115-23	SNORD115-34	SNORD115-43	
SNORD115-14	SNORD115-24	SNORD115-35	SNORD115-44	

The list has been reproduced with permission from supplementary data in Fafard-Couture *et al.* (7).

Beside their canonical function, alternative roles have been described for snoRNAs. Notably, snoRNAs have been shown to interact with messenger RNAs (mRNAs). As examples, *SNORD83B* was found to stabilize the levels of target mRNAs (13), while *SNORD115* was shown to promote alternative splicing of the serotonin receptor 2C (*HTR2C*) (14) and to compete with adenosine deaminases acting on RNA enzymes that otherwise catalyze mRNA editing (15). Furthermore, *SNORD27* was shown to direct splicing of the E2F7 transcription factor (16), and *SNORD88C* was shown to regulate alternative splicing of FGFR3 (17). *SNORD50A* was reported to inhibit 3′ mRNA processing for a subset of transcripts (18). Increasing numbers of reports have identified snoRNAs interacting with proteins. An example is the interaction of snoRNAs and PARP-1, a nuclear enzyme implicated in DNA damage repair. *SNORA73A*, *SNORA73B*, and *SNORA74A* can bind to PARP-1, leading to enhanced ribosome biogenesis, protein translation, and cell proliferation (19). Other alternative roles for snoRNAs include facilitating RNA or DNA methylation by guiding methyltransferases to mRNA or DNA. Accordingly, in previous work, we demonstrated that *SNORD90* and a microdeletion encompassing 2 snoRNAs, *SNORD109A* and *SNORD116*, act by promoting RNA and DNA methylation, respectively (20, 21, 22). *SNORD90* was found to play a role as an m6A RNA methylation inducer. In addition, in a cellular model of induced pluripotent cells differentiated into neurons from this patient with *SNORD116* microdeletion compared to control, we also found differential methylation and expression, especially in the *SLC6A3* gene involved in dopaminergic clearance (21). *SNORD116* microdeletion has been described as part of the minimal region associated with the neurodevelopmental disorder Prader-Willi syndrome (23). Within this region, *SNORD116* is especially interesting because *Snord116* knockout mice present with a decreased expression of the proprotein convertase gene *PCSK1* responsive for prohormone maturation including oxytocin (24), but this was not confirmed in another study (25). Furthermore, research indicates that levels of *Snord116* affect the expression pattern of *Nlgn3* and *Dgkk*. For *Nlgn3*, this effect could be the result of 2 processes: interaction with *Nlgn3* exon 3 in the close vicinity of the splicing site could decrease its usage if it occurred on the pre-mRNA and/or the RNA isoforms that possess a *Snord116* hybridization site may be destabilized by the interaction, as is likely the case for *Dgkk*. Both of these genes are important for brain function (26). In particular, *Nlgn3* is involved in the signaling of oxytocin in dopaminergic cells (27).

### snoRNA in Psychiatric Disorders

The role of snoRNAs has been investigated in several human illnesses (28), such as cancer (29, 30, 31, 32), lung diseases (33), cardiovascular diseases (34), neurological disorders (35), and treatment response (36, 37, 38). In the field of psychiatry, there is already a great deal of interest in ncRNA, particularly microRNA (miRNA) (39, 40, 41, 42, 43). However, to date, relatively few studies have focused on snoRNAs in psychiatric disorders, a surprising fact considering their role and relative importance in the brain. In this review, we summarize the current findings of snoRNAs related to psychiatric disorders and discuss the implications and future directions for this field by using a method described in the Supplement.

### Studies in Autism Spectrum Disorder

Seven studies have investigated an association between snoRNA expression and autism spectrum disorder (ASD). Three of these studies were conducted using blood transcriptomes (44, 45, 46), while the others focused on transcriptomes of postmortem brain tissue (47, 48, 49, 50, 51). A total of 80 different snoRNAs (31 SNORAs and 49 SNORDs) were identified as being associated with ASD in these studies. Among the 80 snoRNAs identified in these studies, 28 were found in more than 1 study, and 4 were detected in both blood and brain tissue (*SNORA26*, *SNORA46*, *SNORA63*, and *SNORA73A*). In blood, *SNORA26*, *SNORDA46*, and *SNORA63* were upregulated in a group of ASD patients with severe symptoms versus mild symptoms (45) while they were downregulated in well-defined samples of the superior temporal gyrus in patients with ASD compared with control participants (51). In blood, *SNORA73B* was upregulated in a group of ASD patients with severe symptoms versus mild symptoms (45), and its expression increased with age in the superior temporal sulcus of participants with ASD (47).

A study used transcriptomic profiling to directly compare laser capture microdissected neurons from anatomically well-defined samples of the superior temporal gyrus, including 59 participants with ASD and 32 control participants who ranged in age from 2 to 73 years. From this analysis, 52 of the 83 neuron-expressed snoRNA genes were upregulated in ASD neurons, and 31 were significantly downregulated, whereas dysregulation of snoRNAs was not observed in bulk tissue. The authors also found that both ribosome and spliceosome components were among the most downregulated in ASD, which is interesting considering the role of snoRNAs in the modification and maturation of rRNAs and small nuclear RNAs (snRNAs). Because snoRNAs and snRNAs are known to be critical regulators of alternative splicing, and splicing alterations are thought to be implicated in ASD and may be observed in the laser capture microdissection–captured neurons, they examined alterations in splicing events associated with snoRNA dysregulation. They identified 835 gene loci in neurons where significant correlations between snRNA expression and intron splicing alterations were observed. Of these 835 loci, 196 were significantly dysregulated in ASD neurons. Several intron clusters correlated with multiple snoRNAs and corresponded to genes involved in synaptic functions. Therefore, they hypothesized that coordinated downregulation of multiple snoRNAs correlate with increased dysregulation of local splicing events in neurons from patients with ASD (51).

### Studies in Schizophrenia

Four studies have explored associations between snoRNA expression and schizophrenia (SCZ). Most of these studies were performed in postmortem brain tissue (50,52,53), whereas only 1 study used blood samples (54). A total of 25 snoRNAs were identified from all these studies, including 5 SNORAs and 20 SNORDs. *SNORA7B* and *SNORD115-10* were found in more than 1 study, with *SNORD115-10* being the only one to be found in both brain and blood. It was found to be upregulated in the anterior cingulate cortex of male patients with SCZ versus female patients (53) and hypermethylated in blood from patients with SCZ versus control participants (54). Interestingly, *SNORD115* and *CRHR1* were previously detected together among 19 genes that were ranked as plausible candidates for psychotic disorder and obesity (55). The *SNORD115* gene (also known as *HBII-52*) was found to regulate the alternative splicing of the 5-HT_2C_ receptor (*HTR2C*) by binding to a silencing element in exon Vb (14). In addition to regulating alternative splicing, *SNORD115* affects RNA editing, an essential mechanism for the generation of *HTR2C* (56), and was shown to inhibit the efficiency of RNA editing of *HTR2C* (57). Moreover, an adverse maternal environment was found to significantly increase the expression of total *Htr2c* in the hippocampi of male mouse offspring associated with increased *Snord115* levels (58). A study found that the firing rate of dopaminergic neurons in the ventral tegmental area and serotonergic neurons in the dorsal raphe nucleus was significantly increased in *Snord115* knockout mice, but this dysfunction was not associated with defects in behavioral abnormalities (binge eating, conditioned place preference for cocaine, or compulsive behavior) (59). Finally, one study challenged these results, finding modest region-specific changes in *HTR2C* RNA editing profiles, while *HTR2C* alternative RNA splicing was unchanged (60).

Ketchesin *et al.* (52) investigated disruptions in sleep and circadian rhythms, which are known to be associated with the onset or exacerbation of psychotic symptoms, in patients with psychosis (including SCZ, schizoaffective disorder, and bipolar disorder with psychosis). They conducted an investigation by performing RNA sequencing and rhythmicity analyses to investigate diurnal alterations in gene expression in human postmortem striatal subregions (nucleus accumbens, caudate, and putamen) in 36 participants with psychosis (primarily SCZ) relative to the same number of control participants. Before this study, they conducted an investigation of participants without psychiatric or neurological disorders and identified snoRNAs as the top rhythmic transcripts in the nucleus accumbens (61). The second study yielded consistent results for control participants, indicating that snoRNAs were among the top 100 rhythmic transcripts in the nucleus accumbens of control participants. However, this enrichment was not observed in SCZ. snoRNAs are known to chemically modify other RNAs, thereby regulating processes such as ribosomal biogenesis, RNA splicing, and RNA editing. The loss of rhythmicity in snoRNAs suggests potential circadian dysregulation of regulation processes associated with snoRNAs such as ribosomal biogenesis, RNA splicing, or RNA editing (52). In addition, snoRNAs were previously found to be involved in diurnal rhythm, and DNA methylation that is responsive to daily rhythms was modified in a mouse *Snord116* deletion model (22).

### Studies in Bipolar Disorder, Major Depressive Disorder, Posttraumatic Stress Disorder, and Suicide

One study indicated that snoRNAs may be associated with bipolar disorder. It examined genotypes and RNA sequencing in brain samples (frontal and temporal cortex) and found that *SNORD3B-2* was upregulated in this population (62).

Three studies have highlighted snoRNAs associated with major depressive disorder (MDD), 2 of which were conducted using brain tissue (63,64) and 1 was conducted using both brain tissue and blood (20). However, no overlap was observed between the results of these studies. Our team quantified snoRNA expression by small RNA sequencing in human postmortem lateral habenula samples of individuals with MDD and psychiatrically healthy control participants, and *SNORA69* showed increased expression in MDD and was technically validated via reverse transcriptase–quantitative polymerase chain reaction. In addition, *Snora69* was upregulated in the mouse lateral habenula and peripheral blood in an unpredicted chronic mild stress mouse model of depression. *SNORA69* guides pseudouridylation onto 5.8S and 18S rRNAs. We quantified the relative abundance of pseudouridines on 5.8S and 18S rRNA in human postmortem lateral habenula samples and found increased abundance of pseudouridines in the MDD group. The relevance of this observation to the psychopathology of MDD remains uncertain. It is possible that changes to rRNA pseudouridylation could affect binding to transfer RNA and mRNA, potentially impacting translation and proteomic profiles in MDD. However, further studies are necessary to confirm this hypothesis (64).

Only one study has investigated posttraumatic stress disorder (PTSD): In a transcriptome-wide expression study in whole blood samples of 324 World Trade Center responders (201 never PTSD, 81 current PTSD, 42 past PTSD), *SNORD54* and *SNORD46* were downregulated in patients with current PTSD compared with World Trade Center responders without PTSD (65).

A genome-wide study examined gene expression in the amygdala, hippocampus, prefrontal cortex, and thalamus in postmortem brain samples from 20 individuals who died by suicide and 7 control participants and found that, in the suicide group, *SNORA13* and *SNORA62* were upregulated in all brain regions; *SNORD114-31*, *SNORD114-15*, and *SNORD114-10* were downregulated in the amygdala and hippocampus; and *SNORA23* was upregulated in the prefrontal cortex (66). Another study performed RNA sequencing in the rostral anterior cingulate cortex of 26 people with depression who died by suicide and 24 matched control participants and found that *SNORD3C* was upregulated in MDD (67).

Therefore, the findings on snoRNA expression in mood disorders, including suicide, are not consistent across reports. This could be explained by differences in the tissues used for analysis. This discrepancy could also be related to the lack of specification of stressful life events, especially early-life stress (ELS). ELS produces dysregulations that occur at different biological levels. Evidence suggests that the hypothalamic-pituitary-adrenal axis and the serotonergic, dopaminergic, neurotrophin, and oxytocin systems may play a role in mediating ELS and MDD (68). Evidence also suggests that ELS induces changes in miRNA function via a complex interaction of genes relevant to the hypothalamic-pituitary-adrenal axis as well as other neuroendocrine signaling systems (69). Therefore, this parameter should also be investigated for snoRNA.

### Studies of Association With Psychiatric Treatment Response

Our team examined snoRNA expression using sequencing data generated from peripheral blood samples collected from 3 independent antidepressant clinical trials involving individuals diagnosed with MDD who were treated with antidepressants (20). *SNORD90* was the only snoRNA consistently upregulated after antidepressant treatment across all 3 independent cohorts. To go further, we investigated *SNORD90* expression in human postmortem anterior cingulate cortex, which was upregulated in individuals who died during a depressive episode but were taking antidepressants. We observed the same pattern of expression in both neuronal and nonneuronal cell types, which suggests that antidepressants do not upregulate *SNORD90* in a cell type–specific manner. The expression of *Snord90* in a paradigm designed to model depressive-like behaviors in mice showed a specific upregulation of *Snord90* in the anterior cingulate cortex of mice that underwent the depression paradigm followed by antidepressant administration, whereas the depression paradigm or antidepressant administration alone did not alter the expression of *Snord90*. In addition, we treated human neuronal cells derived from human induced pluripotent stem cells and differentiated to a monoaminergic phenotype with several psychotropic drugs and observed that *SNORD90* expression was upregulated exclusively by antidepressant drugs. In human neural progenitor cells, we found that overexpressing *SNORD90* resulted in a decrease in NRG3 expression, and knocking down *SNORD90* resulted in an increase of NRG3 expression. We identified a sequence motif for RBM15B, and we hypothesized that *SNORD90* likely acts as a guide RNA for RBM15B and its associated m6A writer complex. We performed m6A immunoprecipitation quantitative polymerase chain reaction and observed an increase in m6A abundance on NRG3 following *SNORD90* overexpression. We knocked down RBM15B expression and found that RBM15B knockdown followed by *SNORD90* overexpression blunted the increase of m6A levels on NRG3. This result supported that *SNORD90* guides m6A modifications onto NRG3. In a mouse model, NRG3 expression was associated with an increase in spontaneous excitatory postsynaptic current frequency following *Snord90* overexpression. This indicated that *SNORD90* mediated downregulation of NRG3 and has implications for glutamate neurotransmission, which translates to behavioral changes such as anxiolytic and antidepressive-like behavior (20).

The snoRNAs associated with psychiatric disorders are summarized in Table 1.

**Table 1:** Summary of snoRNAs Found in Psychiatric Studies

snoRNA	Sample	Psychiatric disorder	Comparison	Change	Reference
SNORA7B	Nucleus accumbens	SCZ	Versus control	Loss of rhythmicity (day versus night)	Ketchesin *et al.* (52)
	Anterior cingulate cortex	SCZ	Male versus female in SCZ	Downregulation	Ragan *et al.* (53)
SNORA11C	Superior temporal sulcus	ASD	Versus control	Upregulation	Ander *et al.* (49)
SNORA13	Blood	ASD	Versus control	Differentially expressed	Kong *et al.* (44)
	Blood	ASD	Severe ASD versus mild ASD	Upregulation	Salloum-Asfar *et al.* (45)
	Amygdala, Hippocampus, Prefrontal cortex, Thalamus	Suicide	Versus control	Upregulation	Glavan *et al.* (66)
SNORA14B	Nucleus accumbens	SCZ	Versus control	Loss of rhythmicity	Ketchesin *et al.* (52)
SNORA21	Blood	ASD	Versus control	Differentially expressed	Kong *et al.* (44)
SNORA22	Superior temporal sulcus	ASD	Old versus young in ASD	Upregulation	Stamova *et al.* (47)
	Primary auditory cortex	ASD	Versus control	Downregulation	Ander *et al.* (49)
SNORA23	Superior temporal gyrus	ASD	Versus control	Downregulation	Zhang *et al.* (51)
	Prefrontal cortex	Suicide	Versus control	Upregulation	Glavan *et al.* (66)
SNORA26	Superior temporal gyrus	ASD	Versus control	Downregulation	Zhang *et al.* (51)
	Blood	ASD	Severe ASD versus mild ASD	Upregulation	Salloum-Asfar *et al.* (45)
SNORA27	Superior temporal sulcus	ASD	Versus control	Upregulation	Ander *et al.* (49)
SNORA28	Blood	ASD	Severe ASD versus mild ASD	Upregulation	Salloum-Asfar *et al.* (45)
	Nucleus accumbens	SCZ	Versus control	Loss of rhythmicity	Ketchesin *et al.* (52)
SNORA40	Superior temporal gyrus	ASD	Versus control	Downregulation	Zhang *et al.* (51)
SNORA41	Blood	ASD	Severe ASD versus mild ASD	Upregulation	Salloum-Asfar *et al.* (45)
SNORA46	Superior temporal gyrus	ASD	Versus control	Downregulation	Zhang *et al.* (51)
	Blood	ASD	Severe ASD versus mild ASD	Upregulation	Salloum-Asfar *et al.* (45)
SNORA48	Superior temporal sulcus	ASD	Old versus young in ASD	Upregulation	Stamova *et al.* (47)
SNORA49	Blood	ASD	Versus control	Differentially expressed	Kong *et al.* (44)
SNORA51	Blood	ASD	Versus control	Downregulation	Salloum-Asfar *et al.* (45)
SNORA52	Blood	ASD	Severe ASD versus mild ASD	Upregulation	Salloum-Asfar *et al.* (45)
SNORA53	Superior temporal gyrus	ASD	Versus control	Downregulation	Zhang *et al.* (51)
	Dorsolateral prefrontal cortex	ASD	Versus control	Differentially expressed	Wright *et al.* (48)
SNORA54	Dorsolateral prefrontal cortex	ASD	Versus control	Differentially expressed	Wright *et al.* (48)
SNORA57	Anterior cingulate cortex	SCZ	Male versus female in SCZ	Downregulation	Ragan *et al.* (53)
SNORA59A	Blood	ASD	Versus control	Differentially expressed	Kong *et al.* (44)
SNORA62	Blood	ASD	Severe ASD versus mild ASD	Upregulation	Salloum-Asfar *et al.* (45)
	Amygdala, Hippocampus, Prefrontal cortex, Thalamus	Suicide	Versus control	Upregulation	Glavan *et al.* (66)
SNORA63	Superior temporal gyrus	ASD	Versus control	Downregulation	Zhang *et al.* (51)
	Blood	ASD	Severe ASD versus mild ASD	Upregulation	Salloum-Asfar *et al.* (45)
SNORA63B	Blood	ASD	Versus control	Downregulation	Salloum-Asfar *et al.* (45)
SNORA67	Blood	ASD	Versus control	Differentially expressed	Kong *et al.* (44)
SNORA69	Blood	ASD	Severe ASD versus mild ASD	Upregulation	Salloum-Asfar *et al.* (45)
	Lateral habenula	MDD	Versus control	Upregulation	Lin *et al.* (64)
SNORA70F	Blood	ASD	Versus control	Differentially expressed	Kong *et al.* (44)
SNORA71D	STS, PAC	ASD	STS versus PAC in ASD	Downregulation	Stamova *et al.* (47)
SNORA73A	Blood	ASD	Severe ASD versus mild ASD	Upregulation	Salloum-Asfar *et al.* (45)
	Superior temporal sulcus	ASD	Old versus young in ASD	Upregulation	Stamova *et al.* (47)
SNORA73B	Anterior cingulate cortex	SCZ	Male versus female in SCZ	Downregulation	Ragan *et al.* (53)
SNORA74A	Dorsolateral prefrontal cortex	ASD	Versus control	Differentially expressed	Wright *et al.* (48)
SNORA74B	Dorsolateral prefrontal cortex	ASD	Versus control	Differentially expressed	Wright *et al.* (48)
SNORA74D	Blood	ASD	Severe ASD versus mild ASD	Upregulation	Salloum-Asfar *et al.* (45)
SNORA76	Superior temporal gyrus	ASD	Versus control	Downregulation	Zhang *et al.* (51)
SNORD1C	Blood	ASD	Versus control	Differentially expressed	Kong *et al.* (44)
SNORD2	Blood	ASD	Severe ASD versus mild ASD	Upregulation	Salloum-Asfar *et al.* (45)
SNORD3A	Blood	ASD	Versus control	Differentially expressed	Kong *et al.* (44)
	Blood	ASD	Versus control	Upregulation	Salloum-Asfar *et al.* (45)
SNORD3B-2	Frontal cortex Temporal cortex	Bipolar disorders	Versus control	Upregulation	Gandal *et al.* (62)
SNORD3C	Blood	ASD	Versus control	Upregulation	Salloum-Asfar *et al.* (45)
	Rostral anterior cingulate cortex	Depressed suicides	Versus control	Upregulation	Zhou *et al.* 2018
SNORD5	Anterior cingulate cortex	SCZ	Male versus female in SCZ	Upregulation	Ragan *et al.* (53)
SNORD6	Superior temporal gyrus	ASD	Versus control	Downregulation	Zhang *et al.* (51)
SNORD7	Blood	ASD	Severe ASD versus mild ASD	Upregulation	Salloum-Asfar *et al.* (45)
SNORD8	Superior temporal gyrus	ASD	Versus control	Downregulation	Zhang *et al.* (51)
	Anterior cingulate cortex	SCZ	Male versus female in SCZ	Upregulation	Ragan *et al.* (53)
SNORD10	Blood	ASD	Versus control	Upregulation	Salloum-Asfar *et al.* (45)
SNORD12	Anterior cingulate cortex	SCZ	Male versus female in SCZ	Upregulation	Ragan *et al.* (53)
SNORD12C	Blood	ASD	Versus control	Differentially expressed	Kong *et al.* (44)
SNORD13	Superior temporal gyrus	ASD	Versus control	Downregulation	Zhang *et al.* (51)
	Primary auditory cortex	ASD	Versus control	Downregulation	Ander *et al.* (49)
SNORD14E	Blood	ASD	Versus control	Differentially expressed	Kong *et al.* (44)
SNORD15A	Blood	ASD	Versus control	Differentially expressed	Kong *et al.* (44)
SNORD17	Dorsolateral prefrontal cortex	ASD	Versus control	Differentially expressed	Wright *et al.* (48)
	Anterior cingulate cortex	SCZ	Male versus female in SCZ	Downregulation	Ragan *et al.* (53)
SNORD22	Blood	ASD	Versus control	Upregulation	Salloum-Asfar *et al.* (45)
SNORD24	Blood	ASD	Versus control	Upregulation	Salloum-Asfar *et al.* (45)
SNORD25	Anterior cingulate cortex	SCZ	Male versus female in SCZ	Upregulation	Ragan *et al.* (53)
SNORD26	Blood	ASD	Versus control	Upregulation	Salloum-Asfar *et al.* (45)
	Anterior cingulate cortex	SCZ	Male versus female in SCZ	Upregulation	Ragan *et al.* (53)
SNORD28	Blood	ASD	Versus control	Differentially expressed	Kong *et al.* (44)
SNORD31	Blood	ASD	Versus control	Differentially expressed	Kong *et al.* (44)
SNORD33	Blood	ASD	Versus control	Differentially expressed	Kong *et al.* (44)
SNORD34	Anterior cingulate cortex	SCZ	Daily use of psychiatric medications	Positive correlation	Ragan *et al.* (53)
SNORD35A	Blood	ASD	Versus control	Differentially expressed	Kong *et al.* (44)
SNORD36A	Dentate gyrus	MDD	Versus control	Upregulation	Mahajan *et al.* (63)
SNORD36B	Blood	ASD	Severe ASD versus mild ASD	Upregulation	Salloum-Asfar *et al.* (45)
SNORD36C	Dentate gyrus	MDD	Versus control	Upregulation	Mahajan *et al.* (63)
SNORD41	Blood	ASD	Versus control	Differentially expressed	Kong *et al.* (44)
SNORD42A	Blood	ASD	Severe ASD versus mild ASD	Downregulation	Salloum-Asfar *et al.* (45)
SNORD43	Blood	ASD	Severe ASD versus mild ASD	Downregulation	Salloum-Asfar *et al.* (45)
SNORD44	Blood	ASD	Versus control	Differentially expressed	Kong *et al.* (44)
SNORD46	Blood	ASD	Severe ASD versus mild ASD	Downregulation	Salloum-Asfar *et al.* (45)
	Blood	PTSD	Versus control	Downregulation	Kuan *et al.* (65)
SNORD47	Anterior cingulate cortex	SCZ	Male versus female in SCZ	Upregulation	Ragan *et al.* (53)
SNORD49A	Anterior cingulate cortex	SCZ	Daily use of psychiatric medications	Positive correlation	Ragan *et al.* (53)
SNORD50A	Blood	ASD	Versus control	Differentially expressed	Kong *et al.* (44)
SNORD51	Blood	ASD	Versus control	Upregulation	Salloum-Asfar *et al.* (45)
SNORD54	Blood	ASD	Versus control	Differentially expressed	Kong *et al.* (44)
	Blood	PTSD	Versus control	Downregulation	Kuan *et al.* (65)
SNORD55	Blood	ASD	Versus control	Differentially expressed	Kong *et al.* (44)
SNORD57	Blood	ASD	Versus control	Differentially expressed	Kong *et al.* (44)
	Blood	ASD	Versus control	Downregulation	Salloum-Asfar *et al.* (45)
SNORD58A	Blood	ASD	Versus control	Differentially expressed	Kong *et al.* (44)
	Anterior cingulate cortex	SCZ	Male versus female in SCZ	Upregulation	Ragan *et al.* (53)
SNORD59A	Blood	ASD	Versus control	Differentially expressed	Kong *et al.* (44)
	Anterior cingulate cortex	SCZ	Male versus female in SCZ	Upregulation	Ragan *et al.* (53)
SNORD60	Blood	ASD	Severe ASD versus mild ASD	Upregulation	Salloum-Asfar *et al.* (45)
SNORD61	Dentate gyrus	MDD	Versus control	Downregulation	Mahajan *et al.* (63)
SNORD64	Superior temporal gyrus	ASD	Versus control	Downregulation	Zhang *et al.* (51)
SNORD65	Blood	ASD	Versus control	Downregulation	Salloum-Asfar *et al.* (45)
SNORD68	Blood	ASD	Versus control	Differentially expressed	Kong *et al.* (44)
SNORD69	Blood	ASD	Versus control	Upregulation	Salloum-Asfar *et al.* (45)
SNORD72	Blood	ASD	Severe ASD versus mild ASD	Upregulation	Salloum-Asfar *et al.* (45)
SNORD73A	Blood	ASD	Severe ASD versus mild ASD	Downregulation	Salloum-Asfar *et al.* (45)
SNORD76	Blood	ASD	Versus control	Differentially expressed	Kong *et al.* (44)
SNORD78	Anterior cingulate cortex	SCZ	Daily use of psychiatric medications	Negative correlation	Ragan *et al.* (53)
SNORD81	Anterior cingulate cortex	SCZ	Male versus female in SCZ	Upregulation	Ragan *et al.* (53)
SNORD83A	Blood	ASD	Severe ASD versus mild ASD	Upregulation	Salloum-Asfar *et al.* (45)
SNORD85	Prefrontal cortex synaptosomes	SCZ	Versus control	Downregulation	Smalheiser *et al.* (50)
SNORD88	STS, PAC	ASD	STS versus PAC in ASD	Upregulation	Stamova *et al.* (47)
SNORD89	Superior temporal gyrus	ASD	Versus control	Downregulation	Zhang *et al.* (51)
SNORD90	Blood	MDD	Treatment response	Upregulation	Lin *et al.* (20)
	Brain	MDD	With and without treatment	Upregulation	Lin *et al.* (20)
SNORD95	Blood	ASD	Versus control	Differentially expressed	Kong *et al.* (44)
SNORD101	Blood	ASD	Versus control	Differentially expressed	Kong *et al.* (44)
	Blood	ASD	Severe ASD versus mild ASD	Upregulation	Salloum-Asfar *et al.* (45)
SNORD102	Blood	ASD	Versus control	Upregulation	Salloum-Asfar *et al.* (45)
SNORD110	Anterior cingulate cortex	SCZ	Male versus female in SCZ	Upregulation	Ragan *et al.* (53)
SNORD111B	Blood	ASD	Severe ASD versus mild ASD	Upregulation	Salloum-Asfar *et al.* (45)
SNORD114-9, 14, 22, 28	Anterior cingulate cortex	SCZ	Male versus female in SCZ	Upregulation	Ragan *et al.* (53)
SNORD114-10	Amygdala, Hippocampus	Suicide	Versus control	Upregulation	Glavan *et al.* (66)
SNORD114-15	Amygdala, Hippocampus	Suicide	Versus control	Upregulation	Glavan *et al.* (66)
SNORD114-23	Dorsolateral prefrontal cortex	ASD	Versus control	Differentially expressed	Wright *et al.* (48)
	Anterior cingulate cortex	SCZ	Male versus female in SCZ	Upregulation	Ragan *et al.* (53)
SNORD114-26	Dentate gyrus	MDD	Versus control	Downregulation	Mahajan *et al.* (63)
SNORD114-31	Amygdala, Hippocampus	Suicide	Versus control	Upregulation	Glavan *et al.* (66)
SNORD115-1	Nucleus accumbens	SCZ	Versus control	Loss of rhythmicity	Ketchesin *et al.* (52)
SNORD115-5	Blood	SCZ	Versus control (monozygotic twins)	Hypermethylation	Melka *et al.* (54)
SNORD115-9	Blood	SCZ	Versus control (monozygotic twins)	Hypermethylation	Melka *et al.* (54)
SNORD115-10	Anterior cingulate cortex	SCZ	Male versus female in SCZ	Upregulation	Ragan *et al.* (53)
	Blood	SCZ	Versus control (monozygotic twins)	Hypermethylation	Melka *et al.* (54)
SNORD115-11, 12, 29, 33, 34, 35, 36, 37, 43	Blood	SCZ	Versus control (monozygotic twins)	Hypermethylation	Melka *et al.* (54)
SNORD115-15, 42	Anterior cingulate cortex	SCZ	Male versus female in SCZ	Upregulation	Ragan *et al.* (53)
SNORD116-3	Blood	SCZ	Versus control (monozygotic twins)	Hypermethylation	Melka *et al.* (54)
SNORD116-4	Anterior cingulate cortex	SCZ	Male versus female in SCZ	Downregulation	Ragan *et al.* (53)
SNORD116-6	Anterior cingulate cortex	SCZ	Daily use of psychiatric medications	Positive correlation	Ragan *et al.* (53)
SNORD116-8, 9, 10, 11	Blood	SCZ	Versus control (monozygotic twins)	Hypermethylation	Melka *et al.* (54)
SNORD116-27, 29	Anterior cingulate cortex	SCZ	Male versus female in SCZ	Upregulation	Ragan *et al.* (53)
SNORD117	Anterior cingulate cortex	SCZ	Daily use of psychiatric medications	Positive correlation	Ragan *et al.* (53)
snoU2_19	Anterior cingulate cortex	SCZ	Male versus female in SCZ	Downregulation	Ragan *et al.* (53)
snoU13	STS, PAC	ASD	STS versus PAC in ASD	Upregulation	Stamova *et al.* (47)
RNU105B	Blood	ASD	Versus control	Differentially expressed	Cheng *et al.* (46)

Details on the samples used, the psychiatric disorder targeted by the study, the type of group compared, and the changes found are presented in the table.

ASD, autism spectrum disorder; MDD, major depressive disorder; PAC, primary auditory cortex; PTSD, posttraumatic stress disorder; SCZ, schizophrenia; STS, superior temporal sulcus.

## Limitations of and Notable Results From the Current Literature

The current review of published studies revealed several limitations. Firstly, only a limited number of studies have reported on the expression of snoRNAs in psychiatric disorders.

Secondly, in instances where multiple studies were conducted on the same disorders, only a limited number of snoRNAs were found to be shared between the studies and/or between different types of samples (blood and brain). Furthermore, these identified snoRNAs were not previously known to be involved in pathophysiological mechanisms associated with psychiatry, with the exception of *SNORD115*, which was identified in various studies and tissue samples regarding SCZ, as previously mentioned.

Another limitation is that studies performed using human postmortem brain tissue are likely to have included participants who were taking medication. This parameter should be taken into account in the analysis of snoRNA expression.

**Figure 2:**
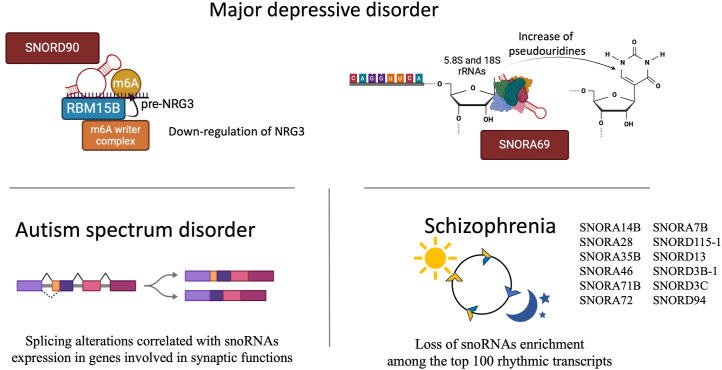
Presentation of the main pathophysiological mechanism identified for snoRNAs as related to psychiatric disorders. These mechanisms involve messenger RNA modifications for major depressive disorder and especially response to antidepressant treatment (20), pseudouridine modifications in rRNA in major depressive disorder (64), dysregulation of snoRNA rhythmic expression in schizophrenia (52), and alteration of messenger RNA splicing of genes related to synaptic functions in autism spectrum disorder (51). rRNA, ribosomal RNA; snoRNA, small nucleolar RNA.

Another limitation of most of the studies reviewed here is that they were not specifically focused on snoRNA expression but rather on large-scale transcriptomic analyses. As a result, they may have lacked the required coverage to study all snoRNAs; also, most of the reports were descriptive, and with a few exceptions illustrated in Figure 2 (20,51,52,64), they did not directly include mechanistic work.

## Future Directions

As a potential future direction, the use of snoRNAs as biomarkers could be considered, as it was previously considered for miRNAs in psychiatry (70,71). However, biomarker research is challenging in psychiatry due to the inaccessibility of sampling brain tissue from patients. One emerging and promising avenue is also the investigation of biomarkers in exosomes, which are a class of extracellular vesicles (EVs) of endocytic origin accessible in biofluids such as saliva, urine, and plasma. EVs can cross the blood-brain barrier and carry cargo and display membrane proteins that allow differentiation and identification of tissue of origin, including brain (72). In the brain, EVs are involved in processes such as synaptic plasticity, neuronal stress response, cell-to-cell communication, and neurogenesis (73). These mechanisms have previously been implicated in psychiatric disorders, leading to the hypothesis that EVs may be involved in psychiatric phenotypes (74,75). EVs contain predominantly shorter RNA species (≤200 nucleotides), including small RNA biotypes (miRNA, snoRNA, snRNA, Y-RNA, vault RNA, and others) (76). Despite the scarcity of data about snoRNAs in this field, some of them have previously been found in exosomes and associated with human diseases. Thus, studies have found a sufficient stability of snoRNA and effect size to consider the use of snoRNA in exosomes as biomarkers. For example, *SNORD3H*, *SNORD1C*, and *SNORA74D* were found as biomarkers of breast cancer in plasma exosomes (77). *SNORD57* and *SNORDB1771* were found to be downregulated during periodontitis and recovered to healthy levels after treatment in plasma exosomes (78). *SNORD115* and *SNORD116* showed high discriminatory power to differentiate Alzheimer’s disease samples from control samples in plasma exosomes (79). Together, these studies demonstrated the stability and abundance of exosomal snoRNAs. This is not surprising because small nucleic acids, such as microRNAs, are known to be very stable (80). There are also a few published data from psychiatric cohorts to assess clinical prediction in this specific field. In addition to the question of stability, it is also a question of abundance and methods used for detection, because for rare RNAs to be discovered in exosomes, the option of deep RNA sequencing should be the gold standard. The increase in sequencing depth and read length allowed by the new technologies will certainly contribute to the emergence of new results.

Most of the studies reported to date looked at snoRNA expression in blood, which did not allow exploration of the pathophysiological hypothesis. Nevertheless, some focused on snoRNA expression in specific brain areas that were chosen because of their implication in the pathophysiology of the disorder. snoRNA expression in the superior temporal sulcus is relevant for ASD because this region supports multimodal integration for social perception and cognition (81). snoRNA expression in the lateral habenula is relevant for MDD because this region plays a role in reward processing, learning, and goal-directed behavior (82), and snoRNA in the anterior cingulate cortex is relevant for SCZ because neurobiological abnormalities of this area have been reported in stages of the disease (83). The regional specificity of these findings brings more accuracy to develop mechanistic hypotheses. In addition, within these specific areas, single-cell or single-nucleus sequencing, which are available in psychiatric research (84), would also allow specification of cell type involvement in the pathology of psychiatric disorders. In addition, more mechanistic studies in animal models are warranted to determine the functional significance of altered snoRNA expression. As shown in the article by Lin *et al.* (20), human and animal approaches appear to be complementary to explore the role of snoRNA in psychiatric disorders. Moreover, these approaches should also integrate sex specificity to explore the therapeutic potential of snoRNAs in psychiatric disorders and their potential as biomarkers for sex specificity in mental health conditions.

Furthermore, previous research investigated how snoRNA expression levels differ between species, reflecting phylogenetic relationships. This area of research could be expanded to provide more details on how snoRNAs have evolved to regulate processes across species, leaving a gap in understanding their evolutionary significance and functional diversification.

Moreover, we have chosen to focus on SNORD and SNORA in this review, but within snoRNA, Cajal body-specific RNAs also play a pivotal role in the pseudouridylation and methylation of snRNAs and rRNAs, thereby influencing their function. Therefore, they may play a relevant role in psychiatric disorders and deserve to be investigated.

Finally, evidence suggests that small RNAs can be posttranscriptionally modified in a variety of ways that significantly influence their function across various biological processes (85,86). Among the possible modifications, *N*-6-methyladenosine (m6A) consists in a methylation modification of the sixth nitrogen (N) atom of adenine (A) (87). It plays a critical role in various biological processes, including meiosis (88,89), circadian rhythm (90), cell differentiation (91), and stress responses (92). Interestingly, snoRNAs were identified as a class of m6A-containing ncRNAs, and over 25% of both classes (SNORA and SNORD) were found to have at least 1 m6A (93). In a subset of human box C/D and C’/D’ snoRNA base pair was found to be reversed by placing a C at the usual G position and thereby creating a GAC sequence that potentially targets the A for N6-methylation. Furthermore, research has identified that methylation in snoRNA prevents binding of the 15.5-kDa protein and the induced folding of the RNA, suggesting a regulatory function for this process (94). Nevertheless, research into the significance and biological impact of small RNA modifications in health and disease remains in its infancy. The study of these modifications may also serve as biomarkers, which could represent a promising avenue for future research (95).

## Conclusions

Studies investigating the pathophysiological mechanisms associated with snoRNA in psychiatric disorders are scarce, although they are warranted. As previously reported for *SNORD90*, snoRNAs may be valuable biomarkers of treatment response or potential targets for new treatments.
