# Supplementary material for: Small Nucleolar RNAs and the Brain: Growing Evidence Supporting Their Role in Psychiatric Disorders
Source: Biol Psychiatry Glob Open Sci. 2024 Nov 10;5(2):100415. doi: 10.1016/j.bpsgos.2024.100415 (PMC11758842; doi:10.1016/j.bpsgos.2024.100415)
Supplement: Document S1 — Supplemental Text [file mmc1.pdf]

SUPPLEMENTARY INFORMATION

Small Nucleolar RNAs and the Brain: Growing Evidence Supporting Their Role in Psychiatric Disorders

Salles *et al.*

Description of the canonical function of snoRNAs and snoRNPs

SnoRNAs canonical function consist in facilitating psuedouridylation and 2'-O-methylation of ribosomal RNAs (rRNA). The rRNA is a molecule in cells that is part of the protein-synthesizing organelle known as the ribosome and is exported into the cytoplasm to help translate the information in messenger RNA (mRNA) into protein. Human rRNAs contain around 200 snoRNA-guided nucleotide modifications. These modifications can enhance ligand interactions and direct three-dimensional folding, thereby fine-tuning ribosome and spliceosome function. Box C/D snoRNAs associate with four proteins: Fibrillarin (FBL)/Nop1p, SNU13(15.5K)/Snu13p, NOP58/Nop58p, NOP56/Nop56p to form snoRNA-ribonucleoprotein complexes (snoRNPs). In addition, FBL is the catalytic component of the Box C/D snoRNP and ensures the deposition of 2′-O methylation of RNA. This is thought to reduce the hydrophilic nature of the nucleotides and allow rRNA to be buried inside of the ribosome. The H/ACA-box snoRNAs (SNORAs) are characterized by the presence of a 3′ (ACA) tail and two hairpin structures connected by a hinge region bearing a conserved (ANANNA, N for nucleotide) motif (also known as the H motif, BOX H). Each hairpin contains an internal loop that forms the psuedouridylation pocket as these regions harbor complementarity sequence to the target RNAs. Box H/ACA snoRNAs associate with four protein co-factors NHP2, NOP10, Gar1, and Dyskerin (DKC)/Cbf5 to form Box H/ACA snoRNPs (Figure 1C). In addition, DCK/Cbf5 is the catalytic component of the H/ACA snoRNP and ensures the conversion of uridine to pseudouridine of RNA. Pseudouridylation can maintain RNA stability and modulate ribosome synthesis. Further, it plays an important role in transforming nonsense codons into sense codons.

Description of the article selection

To conduct it, we searched for articles written in English using the search strategy: ("rna, small nucleolar"[MeSH Terms] OR ("rna"[All Fields] AND "small"[All Fields] AND

"nucleolar"[All Fields]) OR "small nucleolar rna"[All Fields] OR "snorna"[All Fields] OR "snornas"[All Fields]) AND ("mental disorders"[MeSH Terms] OR ("mental"[All Fields] AND "disorders"[All Fields]) OR "mental disorders"[All Fields] OR ("psychiatric"[All Fields] AND "disorders"[All Fields]) OR "psychiatric disorders"[All Fields]) AND ("mental disorders"[MeSH Terms] OR ("mental"[All Fields] AND "disorders"[All Fields]) OR "mental disorders"[All Fields]). A date range was not used since this was an exploratory process. We used a combination of database as recommended (PMID: 29208034). The result consisted of 376 articles from the EMBASE database, 57 articles from Medline, and 506 articles from Google Scholar. We excluded the article focusing on genetic disorders or neurological disorders as well as the article referring only to animal research. We also excluded the review article to focus on original reports. After this filtering, 18 articles were finally retained for further analysis.
